# Impact of Electron Beam (eBeam) Treatment on Meat Quality and Sensory Attributes of Ground Chicken and Turkey

**DOI:** 10.1016/j.psj.2026.107037

**Published:** 2026-05-01

**Authors:** Tanmaie Kalapala, Joaquin Esquivel, Komala Arsi, Anna L.F.V. Assumpcao, Geetha Kumar-Phillips, Ruvindu Perera, Sarah Johnson, Han-Seok Seo, Annie Donoghue, Casey M. Owens, Tomi Obe, Suresh D. Pillai, Palmy R.R. Jesudhasan

**Affiliations:** a Department of Poultry Science, University of Arkansas, Fayetteville, AR 72701, USA; b Poultry Production and Product Safety Research Unit, ARS, USDA, Fayetteville, AR 72701, USA; c Department of Food Science, Center for Food Safety, University of Arkansas System Division of Agriculture, Fayetteville, AR 72704, USA; d Cell and Molecular Biology Program, University of Arkansas, Fayetteville, AR 72701, USA; e Department of Food Science, University of Arkansas, 2650 North Young Avenue, Fayetteville, AR 72704, USA; f National Center for Electron Beam Research, Texas A&M University, College Station, TX 77843, USA

**Keywords:** eBeam, Meat quality, Sensory analysis, Ground chicken, Ground turkey

## Abstract

Microbial contamination of poultry products remains a persistent public health concern, necessitating the adoption of novel intervention technologies to enhance microbial safety while preserving meat quality. However, many intervention strategies aimed at improving microbial safety can adversely affect these quality characteristics. Electron beam (eBeam) is an FDA-approved non-thermal technology that inactivates pathogens by causing irreparable double-stranded DNA breaks. It offers a promising intervention strategy to enhance microbial safety while minimizing heat-induced changes in meat quality. In our previous study, we demonstrated that eBeam dose at 3 kGy achieved 99.99% reduction of *Salmonella* and *Campylobacter* in one-pound commercial packages of ground poultry meat inoculated with these pathogens. In the present study, we evaluated the impact of eBeam treatment at 3 and 4 kGy on the meat quality parameters (pH, cook loss, color, and texture profile) and sensory attributes (including aroma, flavor, basic taste, texture, and feeling factors) of ground chicken and turkey meats compared to untreated controls in commercial ground poultry packages. Statistical analysis was performed using one-way ANOVA followed by Tukey’s post-hoc comparison (P<0.05). Results showed no significant difference in pH at both eBeam doses in ground chicken, whereas in ground turkey, pH was not significantly altered at 3 kGy but was significantly reduced at 4 kGy. Cook loss was significantly reduced in ground chicken following eBeam treatment (P<0.0001), whereas no significant change was observed in ground turkey. Color analysis of raw meats showed that there is a significant reduction in redness (a*) for both eBeam doses in ground chicken (P<0.0001), however, no significant differences were noted in redness (a*), lightness (L*), and yellowness (b*) for ground turkey. Texture profile analysis showed no significant alterations in ground chicken, whereas differences were observed in some texture profile parameters at 4 kGy in ground turkey. Sensory analysis revealed no significant differences in sensory attributes, except for off-notes in the aroma of ground chicken. Overall, eBeam treatment exhibited dose-dependent effects while maintaining meat quality and sensory attributes, with 3 kGy sufficient to achieve microbial safety without compromising product quality.

## Introduction

The global poultry industry has grown significantly over the past few years, driven by increasing consumer demand for high-quality protein sources that are both nutritious and affordable (Castro et al., 2023). Among the currently available poultry products, ground chicken and turkey meat have gained popularity for their versatility, health benefits, and lower costs compared to other species such as beef and pork. Ground meat is a staple in many households and food service settings and is widely used to prepare processed food products, such as sausages, patties, and easy-to-prepare meals. However, ground meat products are highly susceptible to microbial contamination at various points of the processing chain, including slaughter, grinding, packaging, and storage. To address this risk, non-thermal technologies such as eBeam treatment can be used as a post-processing intervention to reduce microbial contamination while maintaining physicochemical and sensory quality attributes of meat products. Among ground meat products, poultry meat is a major vehicle for the transmission of foodborne pathogens, including *Salmonella enterica, Campylobacter jejuni, Escherichia coli, Staphylococcus aureus*, and *Listeria monocytogenes* (Sharma et al., 2025), posing significant food safety concerns. Controlling these pathogens in poultry products remains a significant challenge for the poultry industry and government agencies worldwide (Ayoub et al., 2025). Since ground poultry products are subjected to increased handling during mixing and grinding, there is a greater risk of bacterial growth and cross-contamination, resulting in foodborne illnesses that pose a significant threat to public health (Walter et al., 2025). The consequences of these illnesses not only affect public health but also cause significant economic losses through recalls, product withdrawals, hospitalizations, and reduced consumer confidence. The poultry industry in the United States is facing increasing pressure from regulators and consumers to adopt robust, novel intervention strategies that effectively control foodborne pathogens while maintaining product quality. Current methods used for microbial control in poultry meat are thermal pasteurization, high-pressure processing, chemical sanitization, and refrigeration (Singh et al., 2020). However, these interventions have not sufficiently eliminated foodborne pathogens and are limited by adverse effects on sensory attributes such as texture, color, and flavor, as well as by consumer preference for minimally processed food products free of chemical treatments.

In this context, novel non-thermal technologies are gaining more attention for their ability to improve microbial safety while preserving meat quality. Among these, food irradiation is a well-established, FDA and USDA-approved technology that can effectively reduce pathogens in various food products, including meat and other poultry products (USDA FSIS 2023). The two main types of food irradiation are gamma irradiation, which uses radioactive isotopes such as Cobalt-60, and electron beam (eBeam) technology that uses high-energy electrons produced by an electron accelerator (Pillai and Shayanfar, 2017). Gamma irradiation offers deep penetration, which can aid in the effective treatment of densely packed products. However, gamma irradiation requires longer exposure times to deliver the desired dose for microbial inactivation and is often associated with increased lipid oxidation, which can lead to off-flavor development in poultry meat (Huang et al., 2023; Ahn et al., 2013; Nam and Ahn, 2002b). Additionally, the use of radioactive isotopes presents logistical and regulatory challenges, including security concerns, disposal of isotope sources, and limited acceptance in some countries, which can hinder large-scale adoption in the commercial poultry industry (Praveen et al., 2025).

On the other hand, eBeam technology is a non-thermal process that uses a focused beam of high-energy electrons, causing irreparable DNA single- and double-strand breaks. Hence, it can be used effectively to inactivate pathogens when food products are exposed (Gautam and Venugopal, 2021; Lung et al., 2015). In our previous study, we demonstrated that eBeam doses as low as 2 kGy can effectively reduce pathogenic bacteria like *Salmonella enterica* and *Campylobacter jejuni* in both ground chicken and turkey meat, and with a dose of 3 kGy, we effectively reduced the total aerobic bacteria to below detection limits (Kalapala et al., 2026). Thus, eBeam can be an alternative to gamma irradiation in many ways (Diehl, 2002), including rapid processing, maintaining dose uniformity, no radioactive waste generation, and no residual radioactivity when compared to gamma irradiation, thereby improving the safety, environmental impact, and regulatory compliance (Li et al., 2025; Praveen et al., 2025).

Although the use of irradiation technologies offers many advantages, it often raises concerns about its potential impact on meat quality attributes. Although Gamma irradiation can reduce microorganisms, the physicochemical changes it induces may negatively affect consumer acceptance and product marketability (Hashim et al., 2024). Since eBeam applications are rapid and have no residual effect, we expect minimal or no alteration in meat quality and sensory attributes, which are determinants of consumer acceptance and product marketing (Pillai & Shayanfar, 2018). Meat quality traits such as pH, color, and texture are indicators of freshness, shelf life, and visual appeal (Tolentino et al., 2021; Le Bihan-Duval et al., 2008). Sensory properties, including flavor, aroma, and texture, directly impact eating satisfaction and consumer preferences. Hence, maintaining meat quality while using effective microbial reduction strategies is key to the poultry industry's success. Previous studies have shown that eBeam treatment, when used at optimal doses (e.g., ≤3 kGy), can reduce microbial growth while preserving sensory attributes and quality traits in various meat products such as beef patties (Park et al., 2010), quail (Derakhshan et al., 2018), and sausages (Bouzarjomehri et al., 2020). Although there is growing interest in food irradiation, consumer skepticism about irradiated foods remains a significant barrier to commercial acceptance, driven by misconceptions about safety, taste, and quality. Hence, scientific evidence is required to demonstrate that the sensory and quality attributes of eBeam-treated products are preserved and are similar to those of untreated meat (Kang and Kim, 2025; Bruhn, 1995). Our previous study demonstrated that eBeam treatment at 3 kGy effectively reduced pathogenic and total aerobic bacterial counts (Kalapala et al., 2026); therefore, this dose, along with a higher dose of 4 kGy, was selected in the present study to evaluate meat quality and conduct sensory analysis since the maximum approved dose for meat (refrigerated or unrefrigerated fresh poultry) is 4.5 kGy (Federal Register, 1999). Based on these considerations, this study aims to evaluate the effect of electron beam treatment on the meat quality and sensory attributes of ground chicken and turkey, focusing on color, pH, texture, flavor, odor, and overall acceptability. It is hypothesized that eBeam treatment preserves meat quality without adversely affecting sensory attributes.

## Materials and methods

### Meat sample preparation

All the ground chicken and turkey meat tray packages were obtained from a local grocery store. The weight of the ground turkey package is 0.54 kg, and the ground chicken is 0.45 kg. The depth of the meat package is approximately 2.5-3 cm, and it is packaged with modified atmosphere packaging, securely packed in its original packaging trays at refrigerated temperature and dispatched to the National Center for Electron Beam Research, Texas A&M University, for eBeam treatment, and it is USDA-FSIS approved for commercial eBeam treatment of food and research materials.

### eBeam treatment

The eBeam treatment on meat samples was performed using a high-energy (10 MeV), 15 kW linear accelerator at a dose rate of approximately 3000 Gy/s (Praveen et al., 2025). The samples were treated at 3 kGy and 4 kGy, while untreated (0 kGy) samples were used as the control. Once exposed to the target doses, dosimetry was performed to confirm receipt of the expected dose, as reported by Kalapala et al. (2026). The eBeam irradiation process and dosimetry were performed under both USDA-FSIS- and USDA-APHIS-approved protocols. The eBeam-treated samples and untreated controls were placed in freezing temperature (-20°C) for one week.

### Patties preparation and cooking after eBeam treatment

Frozen meat samples were thawed at 4°C for 24 h to prepare meat patties. Three irradiation treatments were applied, including an untreated control, 3 kGy, and 4 kGy. For each treatment, three meat trays were used, and four patties were prepared from each tray, yielding 12 patties per treatment. The tray served as the experimental unit, resulting in 12 patties (subsamples) for both ground chicken and ground turkey. The meat was manually mixed until a homogeneous consistency was obtained, then placed into plastic petri dishes to form uniform circular patties (87 mm in diameter, 15 mm in thickness, and weighing 110 g), and cooked on a preheated (150°C) 20-inch electric griddle (Model 0705305; National Presto Industries Inc., Eau Claire, WI), flipping every 3 minutes until the core temperature reached 165°F. The internal temperatures of the patties were monitored using a 12-channel Digi sense scanning thermometer (Model 69200-00; Barnant Co., Barrington, IL). Cooked patties were then cooled to room temperature (23°C ± 2°C) before analysis.

### Determination of cook loss

The treated and control patties were weighed before and after cooking to determine the percentage of cook loss using the formula: cook loss (%) = ([raw patty weight – cooked patty weight]/raw patty weight) × 100 (Caldas-Cueva et al., 2021).

### Color and pH analysis

Instrumental color (CIE L* - lightness, a* - redness, and b* - yellowness) was measured in triplicate on the surface of each raw patty before cooking and on the cross-sectional surface of each cooked meat patty using a calibrated colorimeter (Model CR-400; Konica Minolta Sensing Inc., Osaka, Japan). The settings used were illuminant D65 and a 2° observer. The instrument was calibrated against a white tile before measurements were taken. Calibration values were entered according to the Y, x, and y calibration scheme (D_65_) and entered as 84.8, 0.3203, and 0.3378, respectively (Upadhyaya et al., 2022). The pH values of the ground chicken and ground turkey samples were measured in the middle portion of the meat patty using a pH meter equipped with a combination electrode (Model 205, Testo Instruments, West Chester, PA).

### Texture profile analysis

Texture profile analysis (TPA) was conducted at room temperature (23.0 ± 2°C) using a texture analyzer (Model TA. XT Plus; Texture Technologies Corp., Scarsdale, NY). Cylindrical cores (23.0 mm in diameter, 14.4 mm in thickness) were taken from the center of cooked meat patties and subjected to a two-cycle compression test. The samples were compressed to 25% of their initial height using a 5.08 cm cylindrical probe, with the following settings: pretest speed of 1.0 mm/s, test speed of 2.0 mm/s, post-test speed of 2.0 mm/s, load cell capacity of 50 kg, and a trigger force of 5 g. TPA parameters, including hardness, adhesiveness, cohesiveness, springiness, and chewiness, were recorded (Caldas-Cueva et al., 2021).

### Sensory analysis

Descriptive sensory analysis was performed by seven professionally trained panelists at the University of Arkansas Sensory Science Center (Fayetteville, AR). The research protocol of this study was reviewed by the University of Arkansas Institutional Review Board and was determined to be exempt under 45 CFR 46. Each panelist had over 1,000 h of experience evaluating a broad spectrum of food and beverage items, including poultry meat. Lexicon development and training followed the Spectrum^TM^ method (Sensory Spectrum, Inc., Chatham, NJ) as described in previous publications (Lyon and Lyon, 1998; Cavitt et al., 2004; Meilgaard et al., 2007; Morey and Owens, 2017; Kawamura et al., 2025). The lexicon of ground chicken and turkey included sensory attributes such as aroma, flavor, basic taste, texture, and feeling factors, as listed in Table 1, Table 2, respectively. Panelists evaluated the sensory characteristics of ground chicken and turkey meat samples to determine the possible influence of eBeam treatment doses (3 kGy and 4 kGy) on meat quality compared to the control. The samples were removed from the freezer (-20 °C) 24 h prior to sensory analysis, prepared into 100 g meat patties, and then cooked until the internal temperature reached 74°C (165°F) or above as per the USDA recommendation for poultry meat. Test samples were presented in a randomized sequential monadic format. Panelists rated each sample across sensory attributes using a 0-15 scale with 0.1-point increments. To minimize sensory fatigue, at least 5 min were given between sample presentations. During the break, panelists were provided with spring water and unsalted crackers to cleanse their palates. Each sample was evaluated three times by every panelist over three sessions.

**Table 1:** Sensory lexicon developed for eBeam-treated ground chicken attributes by a descriptive sensory analysis panel.

Term	Definition	Technique	Reference
Aroma			
Cooked chicken	Aroma associated with cooked white meats like chicken.	Baked/broiled chicken breast.	Intensities based on universal scale^1^
Brothy	Aroma associated with boiled meat, soup, stock. Weak meaty note.	Chicken stock or soup, beef drippings, stock soup	Intensities based on universal scale
Oily/fatty	Aroma associated with oil/fat	Vegetable oil	Intensities based on universal scale
Blood serum/metallic	Aroma associated with raw lean meat, cooked blood, and serum.	Drippings from raw beef, cooked blood	Intensities based on universal scale
Cardboard	Aroma associated with early stages of oxidation.	Wet cardboard, paper tower	Intensities based on universal scale
Sweet aromatic complex	Aroma associated with products which also have a sweet taste such as molasses, caramelized sugar, maple syrup, honey, and vanilla	Brown sugar, vanilla, caramel	Intensities based on universal scale
Barnyard	Aroma associated with barnyard, silage, wet, dry hay.	White pepper; old casein; processed rotten potatoes	Intensities based on universal scale
Breakfast sausage	Aroma associated with drippings from breakfast sausage.	Breakfast sausage	Intensities based on universal scale
Off-note	Aroma associated with off-notes such as oxidized.		Intensities based on universal scale
Flavor			
Cooked chicken	Aromatics associated with cooked white meats like chicken.	Baked/broiled chicken breast.	Intensities based on universal scale
Brothy	Aromatics associated with boiled meat, soup, stock. Weak meaty note.	Chicken stock or soup, beef drippings, stock soup	Intensities based on universal scale
Blood serum/metallic	Aromatics associated with raw lean meat, cooked blood, serum	Drippings from raw beef, cooked blood	Intensities based on universal scale
Cardboard	Aromatics associated with early stages of oxidation.	Wet cardboard, paper tower	Intensities based on universal scale
Sweet aromatic complex	Aromatics associated with products which also have a sweet taste such as molasses, caramelized sugar, maple syrup, honey, and vanilla	Brown sugar, vanilla, caramel	Intensities based on universal scale
Barnyard	Aromatics associated with barnyard, silage, wet, dry hay.	White pepper; old casein; processed rotten potatoes	Intensities based on universal scale
Breakfast sausage	Aromatics associated with drippings from breakfast sausage.	Breakfast sausage	Intensities based on universal scale
Off-note	Aromatics associated with off-notes such as cardboard, barnyard, and oxidized.	Cardboard, white pepper, oxidized nuts	Intensities based on universal scale
Basic taste			
Salt	The basic taste, perceived on the tongue, stimulated by sodium salt, especially sodium chloride.	Sodium chloride solutions in spring water.	0.2% 2.0 0.35% 5.0
Texture			
Moisture release	The amount of wetness or moistness felt in the mouth after one bite or chew.	Chew sample with molar teeth for up to five chews. (none *→* very juicy)	Carrot^2^ 2.0 Mushroom 4.0 Cucumber 8.0 (chew refs 5 times)
Cohesiveness	The amount the sample deforms rather than splits apart, cracks or breaks	Place sample between the molar teeth and compress fully. May also be done with incisors. (crumbles *→* deforms)	Corn muffin^3^ 1.0 Am. cheese 5.0 Soft pretzel 8.0
Cohesiveness of mass	The amount that the chewed sample holds together.	Chew sample with molar teeth up to 15 times and evaluate them. (loose mass *→* tight mass)	Carrots^4^ 2.0 Mushrooms 4.0 Beef frank 7.5 Am. cheese 9.0
Springiness	The degree to which sample returns to original shape or (2) the rate with which sample returns to original shape.	Place sample between molars; compress partially without breaking the sample structure; release. (no recovery *→* very springy)	Frankfurter^5^ 5.0 Marshmallow 9.0 miniature
Feeling factors			
Astringency	The feeling factor on the tongue or other skin surfaces of the mouth described as puckering or drying	Swish sample in mouth, expectorate and wait 5 seconds. (none → much)	Alum 6.0
Aftertaste	Intensity of the flavor remaining in mouth after swallowing	Measure intensity of flavor remaining in mouth 15 seconds after swallowing (none *→* high)	

1 Soda note in Nabisco Premium Unsalted Tops Saltine Crackers (Nabisco, East Hanover, NJ) = 2.0; cooked apple note in Mott’s Applesauce (Dr. Pepper Snapple Group, Plano, TX) = 5.0; orange note in Minute Maid Frozen Concentrate Orange Juice (Coca-Cola, Atlanta, GA) = 7.5, reconstituted with 1065 mL of filtered water; grape note in Welch’s Concord Grape Juice (Welch’s, Concord, MA) = 10.0.

2 Jiffy corn muffin, cut into ½” cubes (Chelsea Milling Company, Chelsea, MI); American cheese, cut into ½” cubes (Boars Head, Brooklyn, NY); Soft pretzel, cut into ½” cubes (J & J Snack Foods Corporation, Mount Laurel, NJ)

3 Baby carrots, cut into ½” cubes; Button mushrooms, destemmed and cut into ½” cubes; Hebrew National beef frank, boiled for 5 minutes and cut into ½” slices (ConAgra Foods, Indianapolis, IN); American cheese, cut into ½” cubes (Boars Head, Brooklyn, NY)

4 Kraft Foods/Philadelphia ½ in. cube, Cooked 5 min/Hebrew National Beef ½ in. slice, Miniature marshmallow/Kraft Foods 3 pieces, Jello, Knox (see Note) ½ in. cube.

**Table 2:** Sensory lexicon developed for eBeam-treated ground turkey attributes by a descriptive sensory analysis panel.

Term	Definition	Technique	Reference
Aroma			
Cooked poultry	Aroma associated with cooked white meats like chicken, turkey, geese	Baked/broiled chicken/turkey breast.	Intensities based on universal scale^1^
Brothy	Aroma associated with boiled meat, soup, stock. Weak meaty note.	Chicken stock or soup, beef drippings, stock soup	Intensities based on universal scale
Fatty	Aroma associated with fat	Vegetable oil, poultry fat rendered	Intensities based on universal scale
Blood serum/organ/metallic	Aroma associated with raw lean meat, cooked blood, serum.	Drippings from cook blooded, raw beef, chicken liver	Intensities based on universal scale
Cardboard	Aroma associated with early stages of oxidation.	Wet cardboard, paper tower	Intensities based on universal scale
Sweet aromatic complex	Aroma associated with products which also have a sweet taste such as molasses, caramelized sugar, maple syrup, honey, and vanilla	Brown sugar, vanilla, caramel	Intensities based on universal scale
Barnyard	Aroma associated with barnyard, silage, wet, dry hay.	White pepper; old casein; processed rotten potatoes	Intensities based on universal scale
Wet feather	Aromatic associated with wet feather poultry	Lunchmeat, wet feather	Intensities based on universal scale
Breakfast sausage	Aromatic associated with breakfast sausages	Breakfast sausages	Intensities based on universal scale
Off-note	Aroma is associated with off-notes such as oxidized.		Intensities based on universal scale
Flavors			
Cooked turkey	Aromatics associated with cooked white meats like turkey	Baked/broiled turkey breast.	Intensities based on universal scale
Brothy	Aromatics associated with boiled meat, soup, stock. Weak meaty note.	Chicken stock or soup, beef drippings, stock soup	Intensities based on universal scale
Blood serum/organ/metallic	Aromatics associated with raw lean meat, cooked blood, and serum.	Drippings from raw beef, cooked blood, chicken liver	Intensities based on universal scale
Cardboard	Aromatics associated with early stages of oxidation.	Wet cardboard, paper tower	Intensities based on universal scale
Sweet aromatic complex	Aromatics associated with products which also have a sweet taste such as molasses, caramelized sugar, maple syrup, honey, and vanilla	Brown sugar, vanilla, caramel	Intensities based on universal scale
Barnyard	Aromatics associated with barnyard, silage, wet, dry hay.	White pepper; old casein; processed rotten potatoes	Intensities based on universal scale
Wet feather	Aromatics associated with wet feather poultry	Lunchmeat, wet feather	Intensities based on universal scale
Breakfast sausage	Aromatics associated with breakfast sausage	Breakfast sausage	Intensities based on universal scale
Off-note	Aromatics associated with off-notes such as and oxidized.	Oxidized nuts	Intensities based on universal scale
Basic taste			
Salt	The basic taste, perceived on the tongue, stimulated by sodium salt, especially sodium chloride.	Sodium chloride solutions in spring water.	0.2% 2.0 0.35% 5.0
Bitter	The basic taste, perceived on the tongue, stimulated by substance such as quinine, caffein and certain alkaloids	Caffein solutions in spring water.	0.05% 2.0 0.08% 5.0
Texture			
Moisture release	The amount of wetness or moistness felt in the mouth after one bite or chew.	Compress sample with molars one time only. (dry *→* wet)	Carrot^2^ 2.0 Mushroom 4.0 Cucumber 8.0 (chew refs 5 times)
Cohesiveness	The amount the sample deforms rather than splits apart, cracks or breaks	Place sample between the molar teeth and compress fully. May also be done with incisors. (crumbles *→* deforms)	Corn muffin^3^ 1.0 Am. cheese 5.0 Soft pretzel 8.0
Cohesiveness of mass	The amount that the chewed sample holds together.	Chew sample with molar teeth up to 15 times and evaluate them. (loose mass *→* tight mass)	Carrots^4^ 2.0 Mushrooms 4.0 Beef frank 7.5 Am. cheese 9.0
Fibrousness	Amount of grinding of fibers required to chew through the sample	Place sample between molars and chew 3-5 times. Evaluate during chewing (none *→* extremely Fibrous)	Apple^5^ 2.0 Apricot 5.0 Salami 7.0 Celery 9.0
Springiness	The degree to which sample returns to original shape or (2) The rate with which sample returns to original shape.	Place sample between molars; compress partially without breaking the sample structure; release. (no recovery → very springy)	Cream cheese^6^ 0.0 Frankfurter 5.0 Marshmallow 9.0 Miniature
Feeling factors			
Astringency	The feeling factor on the tongue or other skin surfaces of the mouth described as puckering or drying	Swish sample in mouth, expectorate and wait 5 seconds. (none → much)	Alum 6.0
Metallic	The flat chemical feeling factor stimulated on the tongue by metal coins	Bit down on foil	Aluminum foil
Aftertaste	Intensity of the flavor remaining in mouth after swallowing	Measure intensity of flavor remaining in mouth 15 seconds after swallowing (none → high)	

1 Soda note in Nabisco Premium Unsalted Tops Saltine Crackers (Nabisco, East Hanover, NJ) = 2.0; cooked apple note in Mott’s Applesauce (Dr. Pepper Snapple Group, Plano, TX) = 5.0; orange note in Minute Maid Frozen Concentrate Orange Juice (Coca-Cola, Atlanta, GA) = 7.5, reconstituted with 1065 mL of filtered water; grape note in Welch’s Concord Grape Juice (Welch’s, Concord, MA) = 10.0.

2 Baby carrots cut into ½” cubes, button mushrooms, destemmed and cut into ½” cubes, cucumber peeled and cut into ½” cubes.

3 Jiffy corn muffin, cut into ½” cubes (Chelsea Milling Company, Chelsea, MI); American cheese, cut into ½” cubes (Boars Head, Brooklyn, NY); Soft pretzel, cut into ½” cubes (J & J Snack Foods Corporation, Mount Laurel, NJ).

4 Baby carrots, cut into ½” cubes; Button mushrooms, destemmed and cut into ½” cubes; Hebrew National beef frank, boiled for 5 minutes and cut into ½” slices (ConAgra Foods, Indianapolis, IN); American cheese, cut into ½” cubes (Boars Head, Brooklyn, NY).

5 Pink lady apple, peeled and cut into ½” cubes; Mariani apricots, sliced in half (Mariani, Vacaville, CA); Hard salami, cut into ½” cubes (BoarsHead, Brooklyn, NY); Celery, cut into ½” pieces.

6 Kraft Foods/Philadelphia ½ in. cube cooked 5 min/Hebrew National Beef ½ in. slice, Miniature marshmallow/Kraft Foods 3 pieces, Jello, Knox (see Note) ½ in. cube.

### Statistical analysis

Meat tray packages were randomly divided into three treatment groups for ground chicken and ground turkey. Meat quality parameters were analyzed with treatment as the fixed effect and individual patties as the experimental unit, including 12 patties per treatment (4 patties from each of 3 tray packages). Although 4 patties were taken from each tray, individual patties were treated as independent experimental units in the analysis. Descriptive sensory data were analyzed using a mixed model with treatment as the fixed effect, panelist as a random effect, and session as the repeated measure. Data for each parameter were analyzed using one-way ANOVA, followed by Tukey's post hoc test, in GraphPad Prism (version 10 GraphPad Software, San Diego, CA, USA), with P < 0.05 considered statistically significant. Data were analyzed separately for each meat type (ground chicken or ground turkey).

## Results and discussion

### Influence of eBeam treatment on physicochemical properties

**Fig. 1:**
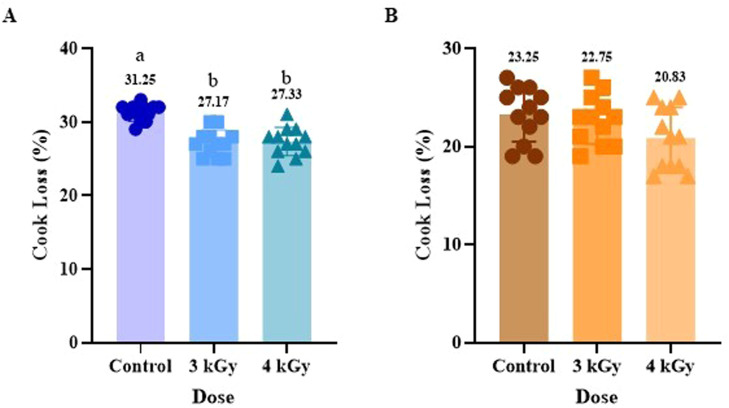
Effect of eBeam treatment on Cook Loss in ground chicken (A) and turkey meats (B). Results represent the mean of samples (Mean ± SD). Bars within a graph with different alphabets differ significantly, P < 0.05, and bars without letters show no significant difference.

**Fig. 2:**
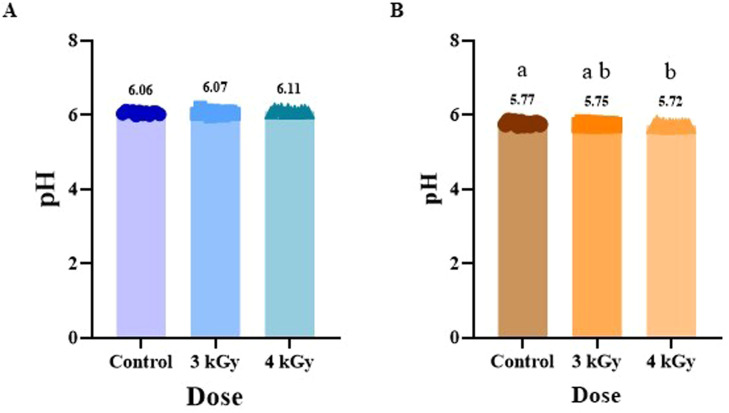
Effect of eBeam treatment on pH in ground chicken (A) and turkey (B) meats. Results represent the mean of samples (Mean ± SD). Bars within a graph with different alphabets differ significantly, P < 0.05, and bars without letters show no significant difference.

The effect of eBeam treatment on the water-holding capacity (WHC) was evaluated by measuring the cook loss and pH of cooked meat patties (Fig. 1, Fig. 2). In ground chicken patties, both eBeam treatments resulted in significantly (P<0.05) lower cook loss than the untreated control, indicating an increase in WHC (Fig. 1 A). However, there is no difference in cook loss between the samples treated with 3 and 4 kGy eBeam doses. This increase in WHC is likely due to structural changes in muscle proteins, specifically myofibrillar proteins, induced by eBeam treatment. The eBeam treatment can induce protein cross-linking or aggregation in a controlled manner, creating a 3-dimensional gel network that traps and immobilizes water molecules, thereby reducing water loss during cooking (Bai et al., 2024). However, previous research evaluating the effect of gamma irradiation on the texture of chicken breast meat showed a significant reduction in WHC due to the damage to muscle fibers and myofibrils, and denaturation of muscle fibers (Huang et al., 2023; Indiarto et al., 2023). This could be due to differences in the source and dose delivery between gamma and eBeam irradiation. Gamma uses radioactive isotopes and delivers a low dose over a longer period, allowing deep penetration, whereas eBeam delivers a high dose in seconds with more limited penetration. At the same dose, both effectively reduce microbial load, but eBeam’s rapid delivery better preserves product quality. However, our results are similar to the positive effects of eBeam treatment at doses ranging from 3 kGy to 5 kGy on the functional characteristics of myofibrillar gels, including improved WHC, as reported by Bai and co-workers (2024).

Unlike ground chicken, ground turkey samples showed no significant difference in cook loss between eBeam-treated samples and untreated controls (Fig. 1 B). This difference between chicken and turkey meat may be due to variations in muscle composition, protein distribution, lipid content, or antioxidant status, which can contribute to the matrix's susceptibility to radiation-induced cross-linking (Indiarto et al., 2023).

In ground chicken meat, pH values did not differ among the treatments, including control, 3 kGy, and 4 kGy (Fig. 2 A). Our results were consistent with previous research, which found that eBeam doses of 2 kGy and 4 kGy did not affect pH in chilled poultry. However, higher doses ranging from 6 kGy to 8 kGy showed a reduction in pH (Uazhanova et al., 2025). On the contrary, eBeam irradiation up to 7 kGy did not alter the pH of frozen duck meat (Arshad et al., 2020). This result also suggests that the increase in the WHC of chicken is not due to a change in pH but can be due to direct structural changes in the meat proteins. In ground turkey, pH was significantly lower in the 4 kGy group compared to the control, while 3 kGy presented similar pH values to the other two treatment groups (Fig. 2 B). This reduction in pH can result from the radiolysis of water, which generates hydrogen ions (H+). These H+ ions are not rapidly neutralized and thus remain in the sample, leading to reduced pH (Kang and Kim, 2025). The change in pH with 4 kGy, but not with 3 kGy, treatment indicates that this change may be dose-dependent. These results suggest that the chemical matrix of ground turkey is different from chicken, and that turkey is more susceptible to radiolytic production of acidic compounds. Also, factors such as irradiation dose, food moisture content, buffering capacity, and the timing of pH measurement may influence the outcome (Kang and Kim, 2025). Despite the slight pH change observed with 4 kGy of eBeam treatment of ground turkey meat, there was no corresponding change in cook loss, suggesting that the shift in pH was not substantial enough to induce structural changes affecting WHC in ground turkey. In summary, eBeam treatment shows species-specific and dose-dependent responses, highlighting the need for targeted, species-specific dose optimization in poultry processing.

### Impact of eBeam irradiation on color parameters

**Fig. 3:**
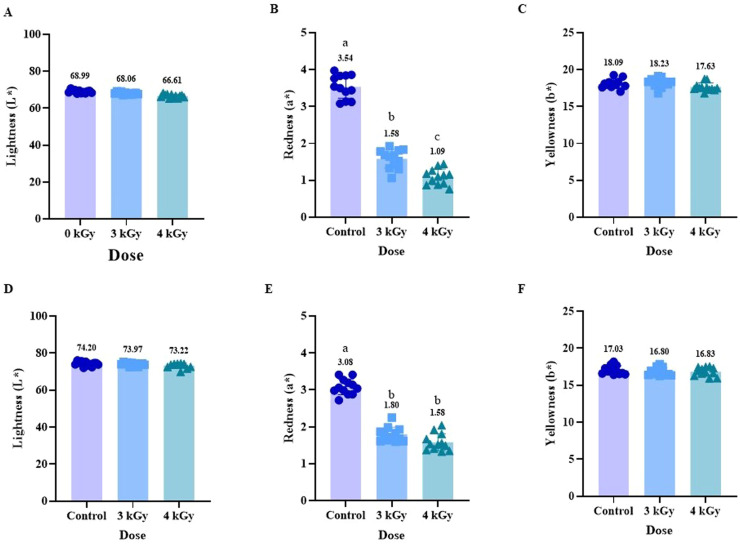
Impact of eBeam treatment on instrumental color analysis L*- lightness (A, D), a*- redness (B, E), and b* - yellowness (C, F) before cooking that were measured on the surface of chicken patties (A, B, C), and internal chicken patties after cooking (D, E, F). Results represent the Mean ± SD of samples and bars with no common letter differ significantly, P < 0.05.

**Fig. 4:**
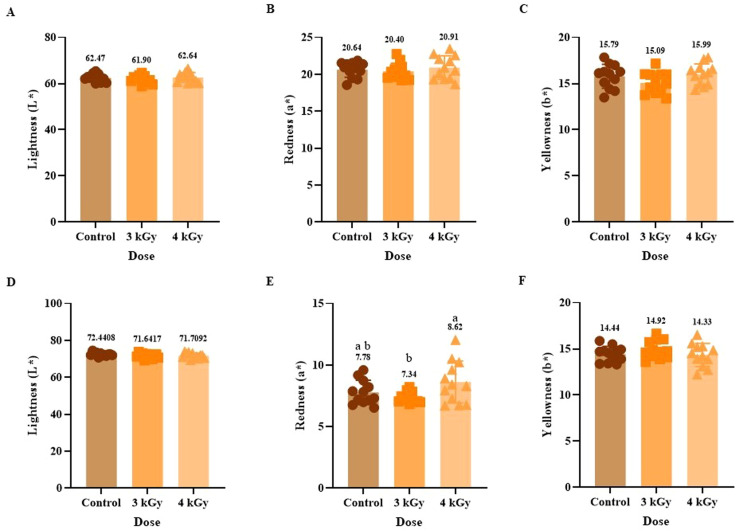
Effect of eBeam treatment on instrumental color analysis L*- lightness (A, D), a*- redness (B, E), and b* - yellowness (C, F) before cooking that were measured on the surface of turkey patties (A, B, C), and internal surface of cooked turkey patties (D, E, F). Results represent the Mean ± SD of samples and bars with no common letter differ significantly, P < 0.05.

The color of meat is one of the most important attributes influencing the acceptance of meat products, and irradiation technologies can alter surface and internal color, depending on species, pigments, and dose level (Tomasevic et al., 2021). The color change induced by irradiation is species-dependent and apparently involves interaction with the heme pigments (Kim et al., 2024). The results of color measurements for ground chicken and turkey are shown in Fig. 3, Fig. 4, respectively. Surface color analysis of each raw patty that was recorded immediately after their preparation showed no difference in lightness (L*) (Figs. 3 A and 4A) or yellowness (b*) (Figs. 3 C and 4 C) in both the meat types. However, a reduction in the redness (a*) of ground chicken was observed in both eBeam treatments compared to the control (Fig. 3 B). In contrast, no differences in a* were observed in ground turkey patties across the treatments (Fig. 4 B). Other researchers also reported a similar reduction of a* without changes in L* and b* values in beef meat treated with UV light (Brugnini et al., 2021). It was previously shown that a decrease in the a* value indicated a decrease in pigment concentration in meat due to myoglobin degradation or denaturation (Ismail et al., 2009). Previous research by Lewis et al. (2002) examined the effect of eBeam treatment on boneless, skinless chicken breasts, in which the a∗ values for irradiated samples increased with dose, suggesting that higher irradiation doses deepen meat color (i.e., more red). A reduction in redness of poultry breast meat has been reported in eBeam irradiated pre-cooked turkey breast meat, with surface color described as grayish brown and a* value decreasing due to myoglobin oxidation (Nam and Ahn, 2002b). A meta-analysis study on the effect of gamma irradiation on chicken meat reported that color changes in irradiated poultry meat can be explained by alterations in muscle pigments, primarily myoglobin and hemoglobin (Asmarani et al., 2024). Since chicken meat has relatively low myoglobin levels and higher hemoglobin levels, it is more prone to oxidative discoloration due to its limited pigment stability and antioxidant capacity (Asmarani et al., 2024; Brewer, 2009). Ionizing radiation generates reactive oxygen species (ROS) and gases such as carbon monoxide (CO), which can interact with the iron molecule in myoglobin to form various pigment complexes (Brewer, 2009; Brito et al., 2011). These reactions may result in either pigment oxidation, producing a brownish appearance, or the formation of stable red pigment complexes, depending on the chemical environment (Ledward, 1970). The oxidation of ferrous (Fe²⁺) iron to ferric (Fe³⁺) iron converts myoglobin to metmyoglobin, leading to discoloration and reduced redness (Brewer, 2004; Asmarani et al., 2024).

The internal color measurements after cooking the patties showed no differences in L* (Figs. 3 D and 4 D) and b* (Figs. 3 F and 4 F) for either chicken or turkey samples across treatments. However, in ground chicken meat, the a* decreased significantly (P < 0.05) at both 3 kGy and 4 kGy compared with the control, with no significant difference between the two eBeam-treated groups (Fig. 3 E). In contrast, in ground turkey meat, a* of the 4 kGy group was increased compared to 3 kGy, while control group values were similar to both eBeam-treated (Fig. 4 E). This may be attributed to the formation of stable red pigment complexes such as CO-myoglobin or NO-myoglobin, generated from irradiation-induced gases and reactive species under reducing conditions. These pigment complexes impart a bright red appearance under reducing conditions, particularly in low-oxygen environments (Brewer, 2004). Previous studies conducted on chicken breast meat using gamma irradiation did not affect L* and b* values, while a* values increased (P < 0.05), especially at a higher dose of 4 kGy, similar to this study. This increase may be due to CO-myoglobin formation during irradiation (Chouliara et al., 2008). The results of this study were consistent with those reported by Nam and Ahn (2002a), who also observed an increase in a∗ values in raw breast turkey meat irradiated at doses of 2.96 and 3.38 kGy. The increase in a∗ value was positively correlated with the amount of CO gas produced and the irradiation dosage. They concluded that the increase in redness of irradiated turkey breast meat was due to the formation of a heme pigment-CO ligand. These findings suggest a difference in the impact of eBeam treatment between ground chicken and ground turkey. This could possibly be due to the difference in fatty acid profile in chicken and turkey meats (Karakök, et al., 2010). Further research has to be conducted to determine the differences in these effects, ground chicken is different from that of ground turkey and is more sensitive to electron beam treatment in terms of redness of meat.

### Impact of eBeam irradiation on instrumental texture profile

**Fig. 5:**
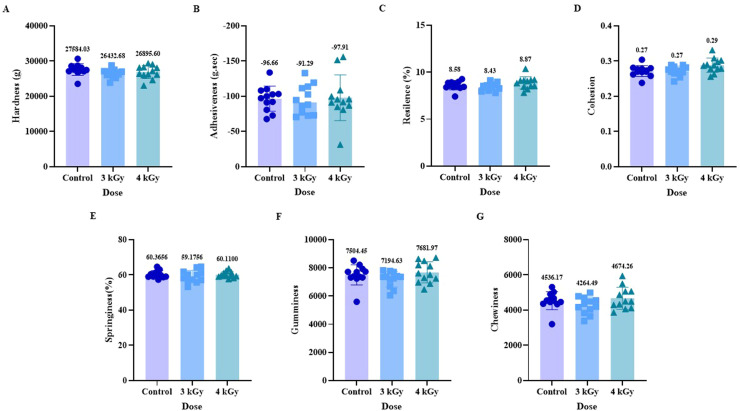
Effect of eBeam treatment on instrumental texture profile analysis on cooked ground chicken patties on texture attributes, including hardness(A), adhesiveness (B), resilience (C), cohesion (D), springiness (E), gumminess (F), and chewiness (G). Each bar includes 12 replicates. Bars with no common letter differ significantly, P < 0.05.

The texture profile attributes (TPA) of the patties provided insight into the physical properties of the patties after eBeam treatment and cooking. In general, any significant change in TPA parameters suggests a change in WHC, likely due to increased protein aggregation or denaturation. For instance, Liu et al., (2022) showed that thermal denaturation of myofibrillar proteins in duck correlated with increases in hydrophobic interactions and measurable changes in texture in TPA. In ground chicken, TPA showed no significant differences in all texture profile attributes, including hardness, resilience, cohesiveness, springiness, gumminess, and chewiness, between the control and eBeam-treated groups, at 3 kGy and 4 kGy (Fig. 5). This preservation of texture quality is a desirable outcome, indicating that the applied eBeam doses achieved the goal of microbial reduction without adversely affecting texture (Kalapala et al., 2026). These results suggest that decreased cook loss and increased WHC did not negatively affect texture attributes, such as increased hardness in the chicken patties, in this study. The meat network structure was stable enough to retain water but not rigid, maintaining the desirable texture of the chicken patties. The resistance of the chicken matrix to eBeam-induced textural alterations is evaluated in minimally processed whole muscle, suggesting that the myofibrillar and connective tissue proteins were not sufficiently degraded or cross-linked by radiolytic products at the applied doses to elicit measurable changes in textural properties (Brewer, 2009). Our results were in contrast to previous research on chicken, which reported that WHC and hardness increased after 5 kGy of eBeam irradiation, while springiness, chewiness, and cohesiveness remained unchanged (Bai et al., 2024). A previous study on chicken breast meat subjected to gamma irradiation showed that decreased hardness, cohesiveness, gumminess, and chewiness could be attributed to denaturation of salt-soluble proteins induced by gamma irradiation (Choi et al., 2015).

**Fig. 6:**
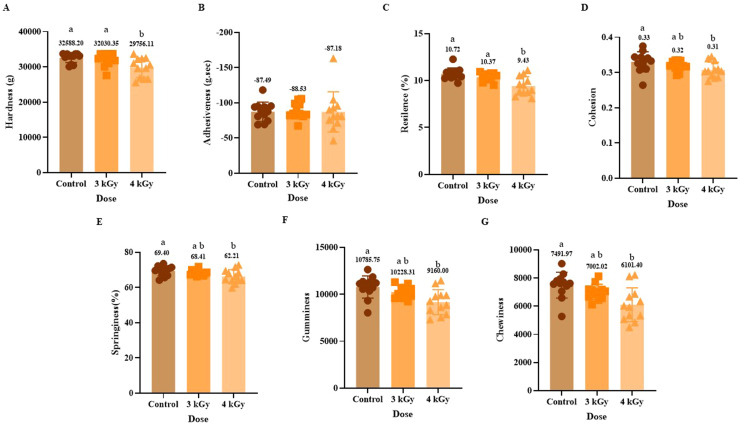
Effect of eBeam treatment on instrumental texture profile analysis on cooked ground turkey patties on texture attributes including hardness(A), adhesiveness(B), resilience (C), cohesion (D), springiness (E), gumminess (F), and chewiness (G). Results represent the mean of samples (Mean ± SD). Each bar includes 12 replicates. Bars with no common letter differ significantly, P < 0.05.

In contrast, the ground turkey samples showed a dose-dependent response in instrumental texture profile analysis (Fig. 6). While the 3 kGy group showed values similar to the control for TPA parameters, the higher dose, 4 kGy, showed significant differences in all measured texture characteristics, including hardness, resilience, cohesiveness, springiness, gumminess, and chewiness, except adhesiveness (Fig. 6). These differences between the 3 kGy and 4 kGy eBeam treatments suggest that a dose threshold exists between these doses, above which the radiolytic effects become more pronounced, producing measurable and significant changes in myofibrillar protein content and texture (Lv et al., 2018). The present results of TPA with 4 kGy of eBeam treatment, coupled with no change in cook loss (Fig. 1 B) and pH reduction (Fig. 2 B), suggest that the radiolytic changes may occur in the turkey matrix at this dose are primarily detrimental to texture and protein structure rather than WHC. These results are supported by previous research done using eBeam irradiation, including turkey breast rolls at 3 kGy (Lee and Ahn, 2005), ready-to-eat (RTE) dry fermented sausages (Cabeza et al., 2009), and RTE cooked ham (Concepción Cabeza et al., 2007) showing no differences in texture parameters. While meats from different animals exhibit varying sensitivities to irradiation doses (Lv et al., 2018). These findings highlight the need for precise, product-specific dose optimization to ensure microbiological safety without compromising the textural quality of meat products.

### Effects of eBeam irradiation on sensory attributes

The sensory properties of meat are essential in determining the overall quality and consumer acceptance (Indiarto et al., 2023). The effects of eBeam treatment on sensory properties of ground chicken and turkey meats were evaluated by a trained panel, and the mean sensory scores for aroma, flavor, basic taste, texture, and feeling factors of eBeam-treated ground chicken and turkey meats are shown in Table 3, Table 4, respectively. The investigation of sensory properties showed that eBeam treatment did not significantly affect the sensory properties of chicken and turkey samples treated at either 3 kGy or 4 kGy. The data show that the eBeam treatment generally did not affect the aroma, flavor, texture, basic taste, or feeling factors of ground chicken and turkey samples. Similar results were reported by García-Márquez et al. (2012), who found that marinated pork loin treated with 3 kGy of eBeam did not alter sensory quality. Also, previous research on ground beef patties irradiated with 2 kGy, 4 kGy, and 6 kGy of eBeam irradiation showed that sensory characteristics were not affected (Tolentino et al., 2021). Although most attributes were not affected, panelists reported an off-note in the aroma of the ground chicken samples at both 3 kGy and 4 kGy. Although the off-note attribute was reported in our descriptive sensory analysis, this has not consistently been associated with decreased consumer acceptance in eBeam-treated poultry products. Previous sensory studies reported no significant differences in overall acceptability or key sensory attributes between eBeam-treated and non-eBeam-treated samples, indicating that the reported sensory differences were detected under descriptive conditions but may not be perceptible to untrained consumers (Johnson et al. 2004, Lewis et al. 2002). In contrast, for the ground turkey samples, off-notes were not detected in either flavor or aroma across treatments. Despite the off-note in aroma reported in chicken samples, all other sensory attributes remained within acceptable ranges for both chicken and turkey across both treatments. Physicochemical changes are critical determinants of sensory profiles in irradiated meat, particularly associated with oxidative pathways. **Lipid peroxidation** can generate volatile secondary metabolites, which correlate with detectable off-aroma (Domínguez et al., 2019). Similarly, **protein denaturation** and oxidation can modify water-holding capacity and myofibrillar fragmentation, directly affecting texture (Zhang et al., 2024). In this study, significant changes in texture parameters were not observed, indicating that the eBeam dose applied did not induce measurable protein related structural modifications.

**Table 3:** Mean comparisons of eBeam-treated ground chicken samples in terms of attribute intensity evaluated by seven trained panelists in descriptive sensory analysis.

Attribute	Control	Treatment 1 (3 kGy)	Treatment 2 (4 kGy)
Aroma			
Cooked Chicken	4.62 ± 0.16	4.33 ± 0.16	4.31 ± 0.16
Brothy	2.69 ± 0.15	2.50 ± 0.18	2.57 ± 0.14
Oily/Fatty	1.75 ± 0.15	1.746 ± 0.14	1.68 ± 0.17
Blood serum/Metallic	1.90 ± 0.19	2.16 ± 0.19	2.23 ±0 .18
Cardboard	1.57 ± 0.13	1.68 ± 0.17	1.82 ± 0.17
Sweet Aromatic Complex	1.67 ± 0.17	1.37 ± 0.15	1.19 ± 0.14
Barnyard	1.96 ± 0.13	2.17 ± 0.17	2.08 ± 0.15
Breakfast sausage	2.41 ± 0.21	1.98 ± 0.24	1.70 ± 0.19
Off-note	0.58 ± 0.13b	1.48 ± 0.25a	1.96 ± 0.25a
Flavor			
Cooked Chicken	6.50 ± 0.23	7.05 ± 0.21	7.087 ± 0.32
Brothy	4.92 ± 0.13	4.72 ± 0.11	5.02 ± 0.13
Blood serum/metallic	3.12 ± 0.19	2.91 ± 0.18	2.93 ± 0.17
Cardboard	2.29 ± 0.18	2.33 ± 0.23	2.27 ± 0.19
Sweet Aromatic Complex	1.62 ± 0.15	1.91 ± 0.22	1.79 ± 0.10
Barnyard	1.58 ± 0.18	1.25 ± 0.16	1.20 ± 0.13
Breakfast sausage	2.12 ± 0.11	2.22 ± 0.14	2.41 ± 0.13
Off-note	2.74 ± 0.28	2.10 ± 0.26	1.91 ± 0.22
Basic taste			
Salt	0.75 ± 0.16	1.31 ± 0.22	1.37 ± 0.24
Texture			
Moisture release	1.75 ± 0.15	1.64 ± 0.11	1.64 ± 0.12
Cohesiveness	3.56 ± 0.16	3.59 ± 0.19	3.54 ± 0.13
Cohesiveness of Mass	3.91 ± 0.22	4.02 ± 0.23	3.74 ± 0.18
Springiness	3.77 ± 0.19	3.72 ± 0.21	3.67 ± 0.19
Feeling factors			
Astringency	3.33 ± 0.13	3.43 ± 0.14	3.33 ± 0.12
Aftertaste	3.75 ± 0.08	3.93 ± 0.07	3.87 ± 0.09

Samples were evaluated in triplicates by trained panelists.

Means ± SEM with the different letters for each attribute are significantly different (P < 0.05) using Tukey's HSD test. Absence of letters denotes no significant differences.

**Table 4:** Mean comparisons of eBeam-treated ground turkey samples in terms of attribute intensity evaluated by seven trained panelists in descriptive sensory analysis.

Attribute	Control	Treatment 1 (3 kGy)	Treatment 2 (4 kGy)
Aroma			
Cooked Turkey	4.38 ± 0.19	4.54 ± 0.20	4.29 ± 0.29
Brothy	2.45 ± 0.14	2.49 ± 0.13	2.18 ± 0.16
Oily/Fatty	1.81 ± 0.11	1.89 ± 0.06	1.71 ± 0.15
Blood serum/Metallic	2.69 ± 0.14	2.28 ± 0.13	2.31 ± 0.21
Cardboard	1.50 ± 0.18	1.56 ± 0.16	1.59 ± 0.17
Sweet Aromatic Complex	1.21 ± 0.18	1.28 ± 0.15	1.19 ± 0.17
Barnyard	1.80 ± 0.10	1.56 ± 0.13	1.63 ± 0.13
Wet Feather	1.36 ± 0.19	1.33 ± 0.17	1.05 ± 0.18
Breakfast sausage	1.02 ± 0.21	1.39 ± 0.19	1.29 ± 0.17
Off-note	0.36 ± 0.18	0.19 ± 0.11	0.10 ± 0.06
Flavors			
Cooked Turkey	4.19 ± 0.28	4.81 ± 0.16	4.55 ± 0.0.27
Brothy	2.35 ± 0.16	2.55 ± 0.14	2.37 ± 0.17
Blood serum/metallic	2.52 ± 0.20	2.67 ± 0.54	2.09 ± 0.14
Cardboard	1.60 ± 0.17	1.67 ± 0.13	1.48 ± 0.15
Sweet Aromatic Complex	1.04 ± 0.14	1.14 ± 0.14	1.17 ± 0.14
Barnyard	1.52 ± 0.13	1.81 ± 0.11	1.76 ± 0.14
Wet Feather	1.14 ± 0.19	1.14 ± 0.16	0.86 ± 0.16
Breakfast sausage	0.93 ± 0.12	1.00 ± 0.17	1.30 ± 0.18
Off-note	0.24 ± 0.12	0.14 ± 0.08	0.14 ± 0.08
Basic taste			
Salt	1.59 ± 0.11	1.76 ± 0.14	1.70 ± 0.16
Bitter	1.13 ± 0.09	1.07 ± 0.09	1.01 ± 0.09
Texture			
Moisture Release	4.82 ± 0.41	4.54 ± 0.24	4.26 ± 0.25
Cohesiveness	4.05 ± 0.23	3.87 ± 0.23	3.61 ± 0.28
Cohesiveness of Mass	4.16 ± 0.20	3.95 ± 0.26	3.77 ± 0.29
Fibrousness	4.09 ± 0.26	3.73 ± 0.15	3.69 ± 0.23
Springiness	3.75 ± 0.17	3.85 ± 0.18	3.64 ± 0.27
Feeling factors			
Astringency	3.66 ± 0.16	3.59 ± 0.15	3.36 ± 0.24
Aftertaste	3.65 ± 0.29	3.46 ± 0.27	3.19 ± 0.25
Metallic	3.23 ± 0.20	3.02 ± 0.17	2.72 ± 0.21

Samples were evaluated in triplicates by trained panelists.

Means ± SEM with the different letters for each attribute are significantly different (P < 0.05) using Tukey's HSD test. Absence of letters denotes no significant differences.

In a previous study on ground beef by Feng et al. (2019), dimethyl disulfide was produced by the radiolysis of methionine, a typical off-odor compound in irradiated meat. In a study by Patterson and Stevenson (1995), gamma irradiation, which produced off-aroma in chicken, was reduced by supplementing the birds’ feed with high levels of α-tocopherol and ascorbic acid prior to slaughter, thereby increasing antioxidant protection in the tissues and reducing the formation of odor-causing volatiles. Incorporating a similar antioxidant-enrichment step as a pre-eBeam strategy will be one of our key future directions to help minimize off-aroma in chicken products. In our study, we used commercially available ground chicken and turkey meats packaged in modified-atmosphere trays, and previous research has shown that this type of packaging can contribute to the development of irradiation-related off-notes in aroma (Nam and Ahn, 2002a). Studies have shown that combining irradiation with natural antioxidants, such as rosemary and oregano extracts, can effectively control microbial growth and maintain the quality of beef burgers during storage (Trindade et al., 2010). Others also reported that vacuum packaging produces fewer of these off-odors (Bagorogoza et al., 2001). As a future direction, we will compare modified-atmosphere and vacuum packaging to determine which approach best minimizes off-aroma formation in eBeam-treated chicken products. We would also explore strategies such as pre-irradiation antioxidants and modified packaging to eliminate off-note in ground chicken.

## Conclusion

In conclusion, this study evaluated the impact of eBeam treatments at 3 kGy and 4 kGy on key meat quality attributes of ground chicken and turkey, including WHC, color, TPA, and sensory properties. The results revealed significant species-specific differences in response to the treatment, underscoring the importance of dose optimization across poultry meat types. An eBeam dose of 3 kGy effectively decontaminated the samples, with minimal changes in physicochemical parameters, color, and texture, and no effect on sensory attributes. Overall, eBeam treatment represents a promising non-thermal intervention for poultry food safety while maintaining the meat quality and sensory attributes. Its industrial applicability will depend on optimizing dose levels, improving economic feasibility, and fostering consumer acceptance through proper labeling, education, and communication.

## CRediT authorship contribution statement

**Tanmaie Kalapala:** Writing – original draft, Visualization, Validation, Methodology, Formal analysis, Data curation, Conceptualization. **Joaquin Esquivel:** Validation, Methodology, Formal analysis. **Komala Arsi:** Writing – review & editing, Visualization, Validation, Project administration, Funding acquisition, Conceptualization. **Anna L.F.V. Assumpcao:** Writing – review & editing, Software, Methodology. **Geetha Kumar-Phillips:** Writing – review & editing, Visualization, Methodology. **Ruvindu Perera:** Writing – review & editing, Validation, Methodology. **Sarah Johnson:** Visualization, Validation, Methodology. **Han-Seok Seo:** Writing – review & editing, Visualization, Validation, Methodology. **Annie Donoghue:** Writing – review & editing, Resources, Funding acquisition. **Casey M. Owens:** Writing – review & editing, Supervision, Methodology, Conceptualization. **Tomi Obe:** Writing – review & editing, Supervision, Methodology, Conceptualization. **Suresh D. Pillai:** Writing – review & editing, Visualization, Resources, Methodology. **Palmy R.R. Jesudhasan:** Writing – review & editing, Validation, Supervision, Software, Resources, Methodology, Investigation, Funding acquisition, Conceptualization.

## Disclosures

All authors have declared that there are no conflicts of interest.
